# MEASURING CLIENT EXPERIENCE IN RESIDENTIAL AGED CARE TO INFORM AND SUPPORT CONSUMER CHOICE

**DOI:** 10.1093/geroni/igz038.1875

**Published:** 2019-11-08

**Authors:** Deirdre M Fetherstonhaugh, Yvonne Wells, Angela Herd

**Affiliations:** La Trobe University, Victoria, Australia

## Abstract

The Australian Government Aged Care Quality and Safety Commission (prior to January 2019 known as the Australian Aged Care Quality Agency) is responsible for accreditation of Australian aged care services which are audited against the Australian Accreditation Standards. Accreditation reports are publicly available. Prior to 2017, some clients were interviewed about their experiences, but the resulting information could not represent the client experience within a service due to low numbers, biased sampling, and an unsystematic approach to asking questions. La Trobe University was engaged to develop and pilot an interview tool to measure client experience for use in accreditation. Potential questions were identified through a literature review, mapped against the Accreditation Standards, and workshopped with an expert reference group. Twenty-four questions and a visual analogue were then piloted. Consumer groups and groups of Indigenous and culturally diverse clients in residential aged care homes were consulted. The perspective of Quality Agency surveyors was sought on the questions’ usability. Statistical analyses sought to identify questions that minimised missing data, were responded to similarly by residents and their representatives, and elicited stable responses on retest. Twelve questions were identified as optimal. The 10 quantitative questions proved to reflect a single underlying dimension (consumer experience) and, when summed and explored through regression analyses, differentiated services significantly. The consumer experience interview tool is now used in all accreditation audits in Australia. Results are then used to generate consumer experience reports, which are published online and can support consumer choice of a residential aged care home.

